# Editorial: Methylation in the human brain

**DOI:** 10.3389/fgene.2025.1589816

**Published:** 2025-04-04

**Authors:** Sheila T. Nagamatsu, Francisco Navarrete, Maria S. Garcia-Gutierrez

**Affiliations:** ^1^ Department of Psychiatry, Yale University School of Medicine, New Haven, CT, United States; ^2^ VA Connecticut Healthcare System, West Haven, CT, United States; ^3^ U.S. Department of Veterans Affairs National Center for Posttraumatic Stress Disorder, Clinical Neurosciences Division, West Haven, CT, United States; ^4^ Instituto de Neurociencias, Universidad Miguel Hernández-CSIC, Elche, Spain; ^5^ Red de Investigación en Atención Primaria de Adicciones, Instituto de Salud Carlos III, Madrid, Spain; ^6^ Instituto de Investigación Sanitaria y Biomédica de Alicante (ISABIAL), Alicante, Spain

**Keywords:** DNA methylation, epigenetics, brain development, neurodegenerative disorders, psychiatric disorders

## 1 Introduction

This Research Topic gathers diverse contributions that aim to elucidate the dynamic patterns of methylation in the human brain and their implications for brain development, function, and neurological disorders. Additionally, we seek to discuss the role of DNA methylation in the human brain, spanning from basic science to disease development, while taking a cross-tissue and cross-population perspective.

The human brain is a complex and enigmatic organ that centers our thoughts, emotions, memories, and behaviors. It is a heterogeneous tissue that rapidly adapts to environmental changes and orchestrates a myriad of functions, from basic physiological processes to advanced cognitive abilities. While the brain undergoes significant growth during prenatal development, it continues to grow throughout childhood and matures during adolescence (Peper et al., 2007). MRI studies have shown that brain volume reaches approximately 95% of adult size by adolescence (Peper et al., 2007). Furthermore, studies reveal changes in white and gray matter volume between the ages of 4 and 20 (Peper et al., 2007).

Although studies have shown a genetic heritability in brain structure and function, it is crucial to consider other contributing components. In this context, regulatory mechanisms that act in the interplay between environmental and genetic factors, such as epigenetics, become highly relevant. Epigenetic mechanisms encompass various biological processes that modify gene expression, including microRNA, histone modifications, and DNA methylation. DNA methylation has been widely studied and is characterized by adding a methyl group to a cytosine base. In most cells, it is usually found at CpG sites, where a cytosine is followed by a guanine. However, specifically in brain tissues, studies have shown the importance of studying non-CpG sites, or CH sites where H can be Adenine, Cytosine, or Timine (Jeong et al., 2021). DNA methylation can occur in different gene regions, including the gene body and promoter.

DNA methylation in promoter regions can readily affect transcription. Studies have shown an association between increased DNA methylation in CpG islands (regions rich in CpG sites) located in the 5′end and gene repression (Van Eijk et al., 2012). To further investigate this association, a study evaluated the relationship between DNA methylation and gene expression in blood samples, identifying both negative and positive associations (Van Eijk et al., 2012). Furthermore, another study suggested that DNA methylation can modulate the relationship between genetic variants and gene expression by evaluating several human cell lines (Zeng et al., 2023). These studies highlight the importance of studying epigenetic mechanisms, especially DNA methylation, by suggesting that they can directly regulate gene expression and indirectly modulate the interplay between genetic factors and gene expression.

Nevertheless, the role of DNA methylation in human brain development and function was recently investigated using whole-genome bisulfite sequencing (Jeong et al., 2021). Neurons and oligodendrocytes from humans, chimpanzees, and rhesus macaques were evaluated after being isolated through fluorescence-activated nuclei sorting (Jeong et al., 2021). Their findings indicate an increase of hypomethylation in CG sites in human prefrontal cortex in neurons and oligodendrocytes. Besides, they identified differential methylated regions enriched for binding motifs for transcriptional factors implicated in cognitive diseases in humans (Jeong et al., 2021). The study also shows increased DNA methylation in CH sites in human prefrontal cortex neurons compared to chimpanzees and rhesus macaques. This increase, coupled with a strong negative correlation between CH methylation and gene expression, suggests that this regulatory mechanism contributed to shaping the transcriptome regulation during evolution (Jeong et al., 2021).

## 2 DNA methylation and neurodegenerative disorders

Epigenetic modifications, in particular DNA methylation, are critical for the onset and progression of neurological and neurodegenerative disorders such as Alzheimer’s disease (AD) or Parkinson’s disease (PD) (Li et al., 2024), which share an aberrant DNA methylation signature characterized by the presence of a deregulated CpG methylation status (Sanchez-Mut et al., 2016). DNA hypermethylation has been reported in the promoters of genes involved in synaptic plasticity, memory formation, and neuroinflammation, which may contribute to cognitive decline and neurodegeneration in AD (Poon et al., 2020). On the other hand, DNA methylation changes in genes related to dopaminergic signaling, mitochondrial function, and neuroinflammation are present in PD (Song et al., 2023).

## 3 DNA methylation and psychiatry disorders

Epigenetics plays a relevant role in the complexity and clinical manifestations of psychiatric disorders, such as mood disorders, schizophrenia, and post-traumatic stress disorder (Grezenko et al., 2023). For example, DNA methylation alterations of oxytocin, closely involved in regulating social behavior, have been found in mental disorders associated with social impairments, such as autism spectrum disorder. Moreover, it is a risk factor for developing pshycopathologies in children exposed to early life experiences (Kraaijenvanger et al., 2019). Here, the study of Danoff et al. establishes the existence of multiple Oxtr transcripts in prairie vole brain and uterine tissue and implicates oxytocin in regulating alternative transcript expression. Only one of the alternative transcripts is associated with DNA methylation in the Oxtr promoter. These data significantly impact our understanding of null mutant models in mice and voles and translation in human birth and behavior. On the other hand, Shastri et al. found no association between the diagnosis of Attention-Deficit/Hyperactivity Disorder (ADHD) and epigenetic alterations at brain and peripheral levels.

## 4 DNA methylation and neurodevelopmental and rare disorders

Interestingly, genome-wide DNA methylation analyses are beginning to identify epigenetic biomarkers that can predict the risk of intellectual/developmental disabilities. In this Research Topic, Kinoshita et al.'s study provides additional insight into this area of research since it demonstrated the loss of NSD2 dysregulated genes related to synaptic transmission and formation through H3K36me2. Another area of interest is the study of epigenetic alterations, particularly in methylation patterns, in the context of rare diseases. Fang et al. supported the potential clinical relevance of exome sequencing for the clinical diagnosis of Joubert and Klinefelter syndromes.

## 5 Future directions and clinical implications

In conclusion, we highlighted the critical role of DNA methylation in the human brain. By exploring epigenetic mechanisms across brain regions, cell types, and populations, we aim to deepen our understanding of the molecular regulation of the brain structure and function. Furthermore, we emphasize the importance of unraveling the complex interplay between environmental and genetic factors, particularly in the context of neurodegenerative, psychiatric, neurodevelopmental, and rare disorders. Ultimately, a better understanding of these regulatory mechanisms will pave the way for translating epigenetic insights into novel diagnostic and therapeutic strategies for these disorders.
